# Predictors of Poor Perinatal Outcome following Maternal Perception of Reduced Fetal Movements – A Prospective Cohort Study

**DOI:** 10.1371/journal.pone.0039784

**Published:** 2012-07-11

**Authors:** Philip J. Dutton, Lynne K. Warrander, Stephen A. Roberts, Giovanna Bernatavicius, Louise M. Byrd, David Gaze, Josh Kroll, Rebecca L. Jones, Colin P. Sibley, J. Frederik Frøen, Alexander E. P. Heazell

**Affiliations:** 1 Maternal and Fetal Health Research Centre, Manchester Academic Health Science Centre, University of Manchester, Manchester, United Kingdom; 2 Health Sciences – Methodology, Manchester Academic Health Science Centre, University of Manchester, Manchester, United Kingdom; 3 Department of Obstetrics, St Mary's Hospital, Central Manchester University Hospitals NHS Foundation Trust, Manchester, United Kingdom; 4 Department of Chemical Pathology, St George's Hospital Medical School, London, United Kingdom; 5 Division of Epidemiology, Norwegian Institute of Public Health, Oslo, Norway; Tehran University of Medical Sciences, Islamic Republic of Iran

## Abstract

**Background:**

Maternal perception of reduced fetal movement (RFM) is associated with increased risk of stillbirth and fetal growth restriction (FGR). RFM is thought to represent fetal compensation to conserve energy due to insufficient oxygen and nutrient transfer resulting from placental insufficiency.

**Objective:**

To identify predictors of poor perinatal outcome after maternal perception of reduced fetal movements (RFM).

**Design:**

Prospective cohort study.

**Methods:**

305 women presenting with RFM after 28 weeks of gestation were recruited. Demographic factors and clinical history were recorded and ultrasound performed to assess fetal biometry, liquor volume and umbilical artery Doppler. A maternal serum sample was obtained for measurement of placentally-derived or modified proteins including: alpha fetoprotein (AFP), human chorionic gonadotrophin (hCG), human placental lactogen (hPL), ischaemia-modified albumin (IMA), pregnancy associated plasma protein A (PAPP-A) and progesterone. Factors related to poor perinatal outcome were determined by logistic regression.

**Results:**

22.1% of pregnancies ended in a poor perinatal outcome after RFM. The most common complication was small-for-gestational age infants. Pregnancy outcome after maternal perception of RFM was related to amount of fetal activity while being monitored, abnormal fetal heart rate trace, diastolic blood pressure, estimated fetal weight, liquor volume, serum hCG and hPL. Following multiple logistic regression abnormal fetal heart rate trace (Odds ratio 7.08, 95% Confidence Interval 1.31–38.18), (OR) diastolic blood pressure (OR 1.04 (95% CI 1.01–1.09), estimated fetal weight centile (OR 0.95, 95% CI 0.94–0.97) and log maternal serum hPL (OR 0.13, 95% CI 0.02–0.99) were independently related to pregnancy outcome. hPL was related to placental mass.

**Conclusion:**

Poor perinatal outcome after maternal perception of RFM is closely related to factors which are connected to placental dysfunction. Novel tests of placental function and associated fetal response may provide improved means to detect fetuses at greatest risk of poor perinatal outcome after RFM.

## Introduction

Despite advances in obstetric care stillbirth remains a significant complication of pregnancy. In some high-income countries including the UK and USA there has been little reduction in stillbirth over the past 20 years [1]. In high income countries the lack of reduction in stillbirths is in part related to the lack of sensitive and specific tests to accurately identify women at highest risk so that intervention may be appropriately directed [2]. One clinical sign intimately related to stillbirth is a reduction in maternally-perceived fetal movements [3]. Intrauterine fetal death (IUFD) is preceded by a reduction in fetal movements (RFM) for over 24 hours in up to 50% of cases [4], [5]. In infants who are alive at presentation, RFM is associated with increased incidence of stillbirth, fetal growth restriction (FGR) and fetomaternal haemorrhage [6], [7]. However, RFM may also occur in non-pathological conditions such as anterior placental site, increased maternal activity and standing position [8], [9]. Even in the absence of formal fetal movement counting 6–15% of women will present in the third trimester with RFM [10], [11]. Tests currently used to assess fetal wellbeing in women with RFM have limited sensitivity to predict fetal compromise and FGR [12]. The combination of the non-specific nature of RFM with a lack of predictive tests has led to wide variation in clinical practice [13], [14].

Further studies are required to determine the most effective screening strategy for women with RFM to identify which women merit more intensive surveillance or delivery to prevent stillbirth. The ability of clinical history and examination to predict poor pregnancy outcome was assessed in a retrospective study of over 200 cases of RFM. In that cohort, the incidence of small for gestational age (SGA) infants was 24% and stillbirth 1.5%; all stillborn infants were severely growth restricted (birthweight <1^st^ centile) [7]. Clinical features found to be predictive of poor pregnancy outcome included: relevant past obstetric history, two or more presentations with RFM and a symphysiofundal height measurement which was small-for-dates on a customised growth chart. However, none of these features were sufficiently sensitive or specific to be of clinical value either alone or in combination [7]. Therefore, better tests are needed to detect infants which are at greatest risk of stillbirth following RFM.

We aimed to identify clinical predictors of infants at greatest risk of stillbirth after maternal presentation with RFM. We aimed to assess maternal clinical variables, ultrasound fetal biometry, liquor volume and umbilical artery Doppler blood flow and measurement of placentally-derived factors in maternal serum. We have recently demonstrated that RFM is associated with abnormal placental structure and impaired placental function [15]. Therefore, we hypothesised that novel strategies to identify placental insufficiency may offer better prediction of poor pregnancy outcome after RFM.

## Materials and Methods

### Patient Recruitment

Ethical approval for the study was obtained from Oldham Research Ethics Committee (08/H1011/83). Women attending the Maternity Day Unit at St Mary's Hospital, Manchester, UK were approached to participate in the study between August 2009 and October 2010 if this was their first presentation with maternal perception of RFM after 28 weeks of gestation in a singleton pregnancy. Women were excluded if the fetus was known to have a structural anomaly, this was a multiple pregnancy, they were unable to give written informed consent or if this was their first contact with the maternity service. A fetal heart trace was performed for 40 minutes to exclude immediate fetal compromise. Provided urgent delivery was not required, written informed consent was obtained and clinical details were recorded from mother's notes including: maternal demographics, details of the duration of RFM, past obstetric and medical history and clinical assessment of symphysiofundal height. The cardiotocograph (CTG) was classified as normal if the baseline rate was 110–160 beats per minute (bpm), the variability was greater than 5 bpm, there were accelerations present and there were no decelerations. If these features were not present the CTG was classified as abnormal [16].

An ultrasound scan was performed to measure estimated fetal weight (calculated from head circumference, abdominal circumference and femur length as previously described [17]), liquor volume and umbilical artery Doppler (Toshiba Xario, 4 MHz probe). A 4.5 mL maternal venous blood sample was obtained by venepuncture into clot-activating gel. Women were then managed according to a standard clinical protocol by their own clinical team who had access to the ultrasound results.

### Pregnancy outcome measures

Following delivery, the pregnancy outcome, mode of delivery and any maternal or neonatal postnatal complications were recorded including: birthweight, Apgar scores, umbilical arterial and venous pH (individual data fields are provided in Table S1). Individualised birthweight centiles were calculated using GROW software (Perinatal Institute, UK, Version 6.4). Poor pregnancy outcome was defined as stillbirth, pre-term birth (defined as delivery before 37 completed weeks gestation), SGA (defined as an individualised birthweight centile <10) or term admission to the neonatal intensive care unit (NICU) for perinatal asphyxia.

### Maternal serum analyses

Serum was obtained from maternal venous blood by centrifugation at 3,000 g for 10 minutes at 4°C; the supernatant was removed at centrifuged at 4,000 g for 10 minutes. 200 µl aliquots of maternal serum were then frozen at −80°C until use. Human chorionic gonadotrophin (hCG) was measured in fresh serum by electrochemiluminescence (Roche E170 analyser, Roche, UK). Lactate dehydrogenase (LDH) activity was assessed by the rate of decrease in NADH (Roche Modular P Unit, Roche, UK). Human placental lactogen (hPL), pregnancy associated plasma protein A (PAPP-A), progesterone and alpha fetoprotein (AFP) were measured by enzyme linked immunosorbent assay (ELISA (Immunodiagnostic systems, Boldon, UK)). Ischaemia modified albumin (IMA) was measured using Albumin Cobalt Binding (ACB®) Test (Inverness Medical Professional Diagnostics Co, USA) and Roche COBAS MIRA® Plus chemistry analyser (Roche Diagnostics, UK) as previously described [18]. The above analytes were selected as they are either produced by the fetus or placenta (AFP, hCG, hPL, PAPP-A, progesterone) or relate to placental ischaemia (IMA, LDH) and dysregulation may indicate fetal or placental compromise. The intra-assay coefficients of variation for all analyses were <5%.

### Placental molecular analyses

To relate the levels of placentally-derived factors to placental structure or function the placenta was collected if women gave birth within 7 days of presentation with RFM. The placenta was collected within 30 minutes of birth and the placenta disc trimmed by removal of cord and membranes and then weighed. Three placental samples were taken, one from the centre of the disc, another from the edge and one from the middle of these two points. The tissue was washed, treated with RNA later (Ambion, UK) and snap frozen at −80°C. Villous tissue from each region was pooled and RNA extracted using TRI reagent (Sigma-Aldrich, Poole, UK). Contaminating genomic DNA was removed by treatment with DNAse (DNA-free kit, Ambion). RNA purity was verified by UV spectroscopy and quantified using Ribogreen (Invitrogen, UK). cDNA was reverse transcribed from 250 ng RNA by AffinityScript cDNA synthesis kit in duplicate reactions to overcome inherent variability (Agilent, UK). Real time PCR for housekeeping gene YWHAZ was performed on cDNA replicates using the following primers: forward – CCTGCATGAAGTCTGTAACTGAG, reverse – TTGAGACGACCCTCCAAGATG. Forty cycles of PCR were performed using an MX3000/3005P (Agilent) thermal cycler with an initial enzyme activation and template denaturation for 10 minutes at 95°C, followed by 30 s at 95°C, 60 s annealing at 60°C and an extension phase for 60 s at 72°C followed by melt curve analysis. mRNA expression was quantified using SYBR Green I and dissociation curve analysis was performed to ensure amplification specificity. A standard curve was constructed from serially diluted placental reference cDNA (Agilent) to interpolate the quantities of cDNA from the Ct values. cDNA replicates were pooled if the %CV for YWHAZ was within 25%, ensuring a good representation of the original mRNA content. The mRNA expression of hCG, hPL and 3β-hydroxysteroid dehydrogenase (3β-HSD, enzyme responsible for progesterone biosynthesis) were determined by real-time PCR as above using the following primer sequences: hCG forward – TCACTTCACCGTGGTCTCCG, reverse – TGCAGCACGCGGGTCATGGT; hPL forward – CATGACTCCCAGACCTCCTTC, reverse – TGCGGAGCAGCTCTAGATTG; 3β-HSD forward – TAACGGGTGGAATCTGAAAAACG, reverse CTAGCAGAAAGGAATCGGCTTC. The mRNA expression of the genes of interest (hCG, hPL and 3βHSD) were normalized to YWHAZ.

The power calculation for the sample size was based on the assumption that patients who had poor pregnancy outcome would have similar blood levels of placentally-derived proteins to those described in FGR [19]. To detect a difference in hPL with α = 0.9, we would require 10–24 patients to have poor pregnancy outcome. A conservative estimate was made that 10% of women presenting with RFM will have a poor outcome. Therefore, we aimed to recruit 300 participants.

Statistical analysis was performed using R (R Foundation for Statistical Computing, Austria). Continuous variables were compared using Mann-Whitney U test or Students' t-test dependent on the distribution of data. Where data were not normally distributed, the variables were logarithmically transformed prior to logistic regression. Logistic regression was used to quantify the effects of potentially prognostic variables on poor outcome, both univariate models and models adjusting for previously described predictors of poor outcomes (gestation, diastolic blood pressure, estimated fetal weight centile, liquor volume and cigarette smoking) were fitted in addition to other statistically significant factors in univariate analysis (abnormal CTG, number of fetal movements normalised to 45 minutes).

## Results

### Participant demographics and history of RFM

**Figure 1:**
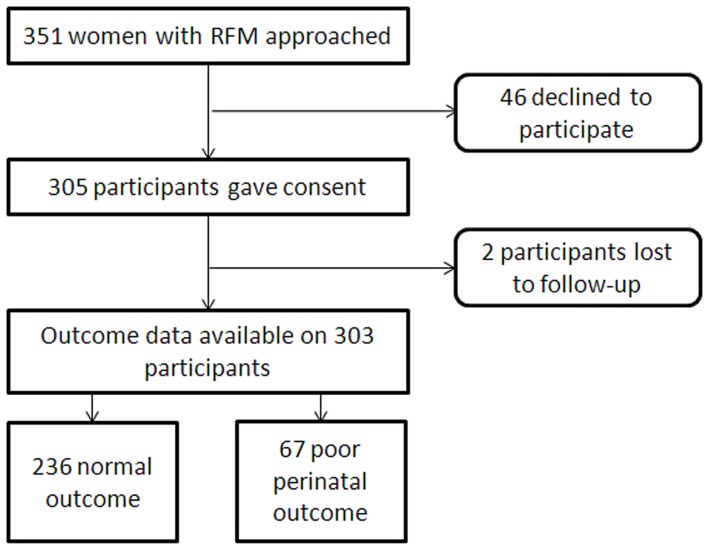
Flow diagram describing recruitment and progress of participants to the cohort study.

During the study period 7,651 women gave birth at the participating unit. 351 women with RFM (4.6%) met the inclusion criteria and were approached to participate in this study. Forty-six women declined and 305 women gave written consent (Figure 1). Of the 305 women who participated in the study, 2 were lost to follow-up. This gave a total of 303 women with complete outcomes. There were no significant differences in maternal age, BMI, gravidity or parity between participants and non-participants (Table 1). Women who participated in the study were more likely to be of European ethnic group, to have presented at an earlier gestation and to have a longer duration of RFM and perceived absent fetal movements than those who did not participate (Table 1). Despite these differences between participants and non-participants women entering the study came from a variety of ethnic groups, had a wide age range and were recruited from 28^+0^ to 42^+0^ weeks gestation. There was no significant difference in pregnancy outcome in women who participated in the study and those who declined (Table 1).

**Table 1:** Demographic characteristics of women participating or not consenting to participate in the study.

Maternal Characteristic	Participants recruited (n = 305)	Non-consenters (n = 46)	p value
Age	28 (17–46)	28 (18–43)	0.77
BMI	25.2 (17.2–52.8)	24.0 (18.1–44.1)	0.20
Gravidity	2 (1–9)	2 (1–8)	0.97
Parity	1 (0–7)	1 (0–4)	0.79
Ethnicity			
*Bangladeshi*	4 (1.3%)	5 (10.9%)	p<0.001
*Black African*	29 (9.5%)	4 (8.7%)	
*Black Caribbean*	17 (5.6%)	3 (6.5%)	
*Chinese*	3 (1.0%)	1 (2.2%)	
*European*	171 (56.1%)	7 (15.2%)	
*Indian*	9 (3.0%)	3 (6.5%)	
*Middle Eastern*	15 (4.9%)	3 (6.5%)	
*Mixed Ethnicity*	16 (5.2%)	1 (2.2%)	
*Pakistani*	38 (12.5%)	18 (39.1%)	
*South East Asian*	3 (1.0%)	1 (2.2%)	
Cigarette smokers	43 (14.1%)	Not recorded as declined to participate	N/A
Gestation at Presentation (weeks^+days^)	36^+2^ (28^+0^–42^+0^)	38^+3^ (28^+6^–41^+2^)	0.003
Duration of RFM (hours)	48 (3–1690)	24 (5–168)	0.001
History of absent fetal movements	110 (36.1%)	7 (21.7%)	0.004
Duration of absent fetal movements (hours)	7 (2–96)	14 (10–48)	0.86
Average number of movements in 45 minutes	9 (0–72)	Not recorded as declined to participate	N/A

Pregnancy Outcome	Participants (n = 303)	Non-consenters (n = 40)	p value
Gestation at delivery (weeks^+days^)	40^+1^ (29^+0^–42^+4^)	40^+2^ (38^+0^–41^+4^)	0.36
Birthweight (g)	3380 (850–5080)	3240 (2480–3940)	0.21
Birthweight centile	34 (0–100)	39 (3–95)	0.85

The median reported duration of RFM at presentation was 48 hours, although there was a wide range from 3 hours to over 1 week; 36.1% of participants reported absent fetal movements for a period of time. When women were on the fetal heart rate trace for 45 minutes, the median number of movements recorded was 9, ranging from 0 to 72. At the time of presentation with RFM, a clinical history was taken. Forty-three women (14.1%) reported smoking cigarettes, ranging from 2–20 cigarettes/day, 7 (2.3%) women reported drinking alcohol, ranging from 1–6 units per week. Thirty-six women (11.8%) had significant past medical history including: anaemia, asthma, epilepsy, hypothyroidism, hypertension and thrombophilia. Fifteen of the 157 (9.6%) parous women had significant past obstetric history including: FGR, placental abruption, preeclampsia, preterm birth, stillbirth and SGA infants. Measurement of maternal blood pressure found 5 participants were hypertensive with a blood pressure >140/90 mmHg and two participants had significant proteinuria (≥2+) on urine dipstick. Twelve women (4%) had a non-reassuring fetal heart rate trace, in four women these changes became pathological necessitating emergency Caesarean section. At ultrasound scan, 1 case had absent end-diastolic flow detected by umbilical artery Doppler, 14 cases had oligohydramnios and 29 cases had an estimated fetal weight ≤10^th^ centile.

### Pregnancy outcome in women with RFM

Two hundred and thirty-six participants (77.9%) had a normal outcome compared to 67 who had a poor outcome (22.1%). Of those with poor outcome, 7 were preterm (with birthweight >10^th^ centile, 4.1%), 2 term infants were admitted to NICU (0.7%), and 51 (16.8%) were SGA at term and 7 (2.3%) were SGA preterm (Table 2). Although there were no stillbirths in this cohort, 4 participants underwent emergency Caesarean section for pathological CTG, intrauterine asphyxia was confirmed at delivery by acidaemia in the umbilical arterial sample.

**Table 2:** Pregnancy outcome in women presenting with reduced fetal movements.

Pregnancy Outcome	Number	%
Normal	236	77.9
Preterm (normal weight)	7	2.3
Small for gestational age (Preterm)	7	2.3
Small for gestational age (Term)	51	16.8
NICU admission for Perinatal Asphyxia	2	0.7
Total	303	100

Small for gestational age was defined as a birthweight less than 10^th^ centile.

### Placentally-derived factors in maternal serum

**Figure 2:**
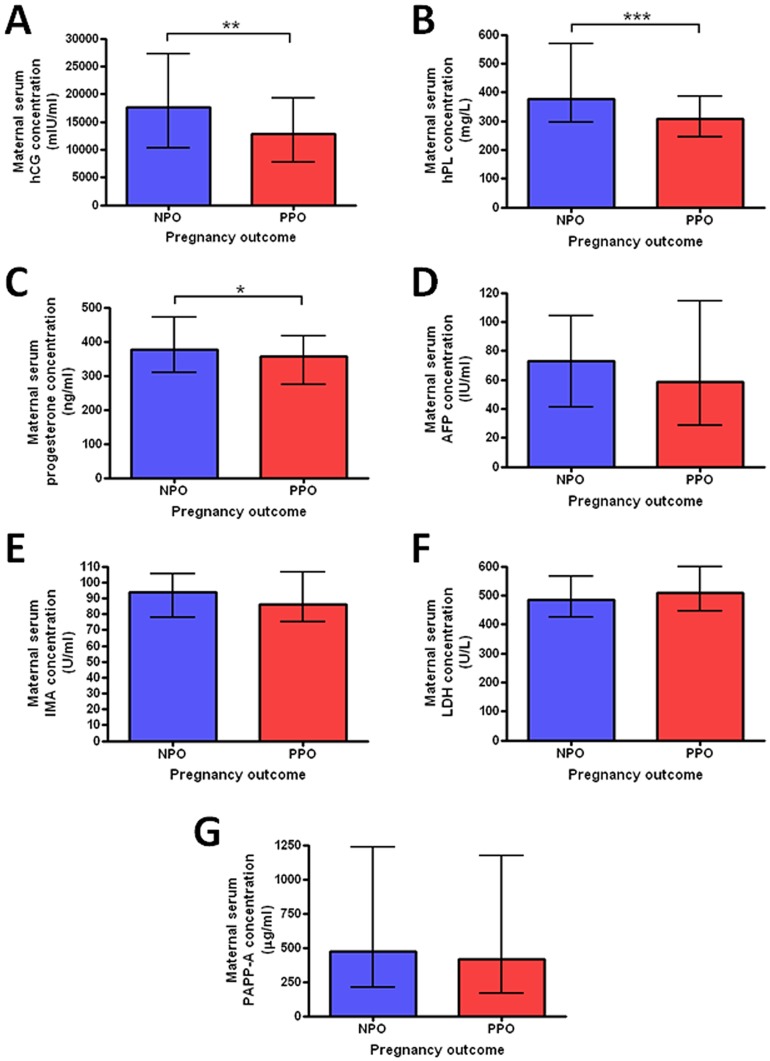
Placentally-derived or modified factors measured in maternal serum. A) human chorionic gonadotrophin (hCG), B) human placental lactogen (hPL), C) progesterone, D) alphafetoprotein (AFP), E) Ischemia-modified albumin (IMA), F) Lactate dehydrogenase (LDH) and G) Pregnancy-associated plasma protein A (PAPP-A). * p<0.05, ** p<0.01, ***p<0.001.

**Figure 3:**
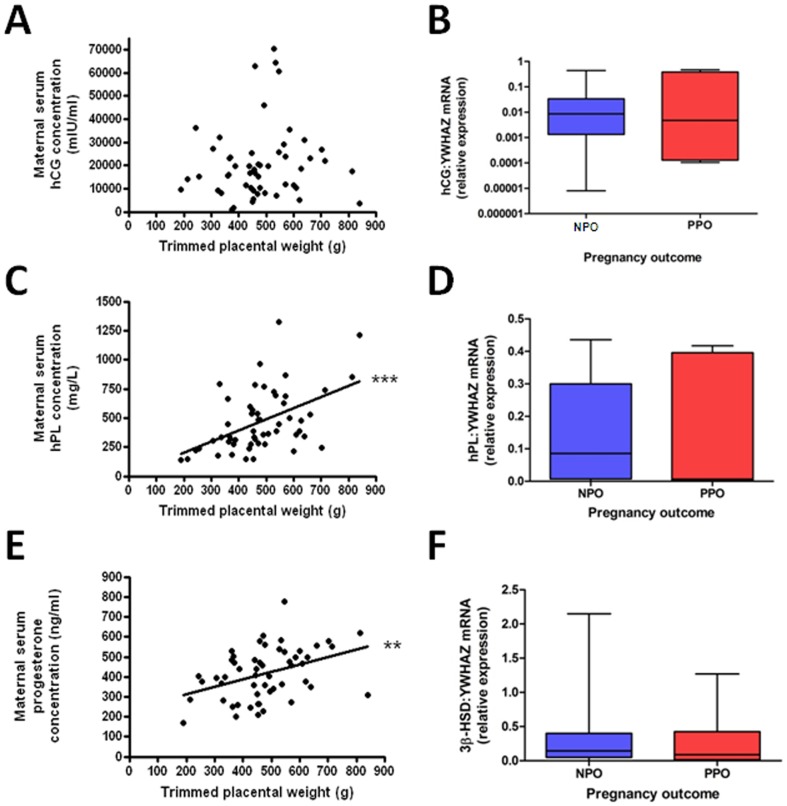
Relationship between placentally derived factors, placental weight and mRNA expression. A) serum hCG was not related to placental weight, B) There was no difference between hCG mRNA and pregnancy outcome, C) serum hPL positively correlated to placental weight (*** p<0.001), D) There was no difference between hPL mRNA expression and pregnancy outcome, E) serum progesterone positively correlated to placental weight (** p<0.01), F) There was no difference between 3β-HSD mRNA expression and pregnancy outcome.

The concentrations of hCG, hPL and progesterone were significantly lower in women who had poor perinatal outcome following RFM compared to those with normal outcomes. There was no difference in AFP, IMA, LDH or PAPP-A between women with poor perinatal outcome and those with normal outcomes (Figure 2). To determine whether maternal serum levels of hCG, hPL and progesterone were related to placental mass and/or placental synthetic function further analyses were conducted. Placental tissue was collected from 55 women who gave birth within one week of the serum sample being obtained. There was a significant positive correlation between placental weight and hPL (p<0.001) and progesterone (p<0.01), but this relationship was not seen with hCG (Figure 3). Real-time PCR for placental transcripts of hPL, hCG and 3β-HSD, the key-synthetic enzyme for progesterone, did not reveal any significant difference between placentas from pregnancies ending in poor perinatal outcome compared to those with normal outcome (Figure 3).

### Predictors of Pregnancy Outcome after RFM

Univariate logistic regression demonstrated a significant relationship between poor pregnancy outcome and the number of movements felt during the fetal heart rate trace, abnormal fetal heart rate trace, diastolic blood pressure, estimated fetal weight centile, liquor volume, log [hCG] and log [hPL] (Table 3). Several other variables approached statistical significance including: cigarette smoking (p = 0.062) and systolic blood pressure (p = 0.078). Other clinical characteristics including significant past medical history and past obstetric history did not show a statistically significant relationship with pregnancy outcome.

**Table 3:** Crude and Adjusted Odds Ratios* for prediction of poor pregnancy outcome after presentation with RFM.

Variable	Median (range) or *n*	Crude Odds Ratio	Confidence Interval	p value	Adjusted Odds Ratio*	Confidence Interval	p value
Gestation at presentation (days)	254 (196–294)	1.00	0.99–1.01	0.29	**0.99**	**0.97–0.99**	**0.026**
Gestation at presentation <37 weeks	*n = 172*	0.97	0.92–1.01	0.14	**0.95**	**0.90–1.00**	**0.048**
Maternal Age	28 (17–46)	1.00	0.95–1.05	0.95	1.04	0.98–1.10	0.24
Maternal BMI	25.2 (17.2–52.8)	0.98	0.94–1.03	0.46	1.01	0.95–1.08	0.71
Gravidity	2 (1–9)	0.93	0.77–1.12	0.43	0.97	0.77–1.23	0.82
Parity	1 (0–7)	0.94	0.72–1.13	0.63	0.99	0.72–1.37	0.95
Smoking (Yes/No)	*n = 43*	1.96	0.96–4.00	0.06	1.55	0.55–3.68	0.32
Alcohol (units/week)	0 (0–6)	0.77	0.31–1.87	0.56	0.72	0.19–2.68	0.62
Duration of RFM (hours)	48 (3–1690)	1.00	1.00–1.01	0.55	1.00	0.99–1.00	0.55
Significant Medical History (Yes/No)	*n = 36 (11.8%)*	1.01	0.44–2.33	0.99	0.88	0.32–2.46	0.81
Significant Obstetric History (Yes/No)#	*n = 15 (9.6%)*	1.35	0.40–4.53	0.63	1.61	0.36–7.35	0.54
FMs normalised to 45 minutes	9 (0–72)	**0.96**	**0.92–0.99**	**0.022**	0.98	0.95–1.02	0.28
Abnormal CTG (Yes/No)	*n = 12*	**8.04**	**2.34–27.62**	**0.001**	**7.08**	**1.31–38.18**	**0.02**
Further episodes of RFM (Yes/No)	*n = 69*	1.15	0.91–1.46	0.24	1.03	0.75–1.42	0.86
Systolic blood pressure (mmHg)	110 (80–150)	1.02	1.00–1.04	0.078	1.00	0.96–1.04	0.92
Diastolic blood pressure (mmHg)	60 (50–114)	**1.05**	**1.01–1.08**	**0.004**	**1.04**	**1.00–1.09**	**0.045**
Estimated fetal weight centile	53 (0–100)	**0.95**	**0.94–0.97**	**<0.001**	**0.95**	**0.94–0.97**	**<0.001**
Amniotic fluid index (cm)	125 (10–252)	**0.99**	**0.99–0.99**	**0.019**	0.99	0.98–1.00	0.13
Maximum pool depth (cm)	51 (0–133)	**0.97**	**0.96–0.99**	**0.002**	0.99	0.96–1.01	0.28
Abnormal Umbilical Artery Doppler	*n = 1*	NA†	NA†	NA†	NA†	NA†	NA†
Umbilical Artery Pulsatility Index	0.8 (0.5–1.4)	3.29	0.75–14.42	0.12	2.96	0.38–23.21	0.30
Log hCG	4.20 (2.85–5.09)	**0.67**	**0.48–0.92**	**0.015**	0.88	0.32–2.46	0.81
Log hPL	2.54 (2.15–3.12)	**0.23**	**0.11–0.47**	**<0.001**	**0.13**	**0.02–0.99**	**0.05**
Log IMA	1.97 (1.42–2.14)	0.60	0.22–1.61	0.31	0.53	0.04–7.91	0.65
Log LDH	2.70 (2.36–2.94)	2.67	0.70–10.17	0.15	5.42	0.11–278.74	0.40
Log PAPP-A	2.67 (1.63–4.52)	0.94	0.77–1.14	0.50	0.78	0.44–1.38	0.39
AFP	70.9 (1.7–232.2)	0.99	0.99–1.00	0.23	0.99	0.99–1.00	0.13
Progesterone	369.1 (167.9–777.8)	0.99	0.99–1.00	0.079	1.00	1.00–1.00	0.73

* Adjusted for gestation, number of fetal movements normalised to 45 minutes, abnormal CTG, diastolic blood pressure, estimated fetal weight centile, maximal pool depth and smoking status.

# only calculated for parous participants.

† Odds ratios could not be calculated as only one infant had abnormal umbilical artery Doppler at initial assessment.

When multivariate regression was performed, adjusting for previously reported predictors of fetal wellbeing, the following factors remained predictors of poor perinatal outcome: diastolic blood pressure, estimated fetal weight centile and log [hPL]. Of the 67 poor perinatal outcomes, 4 were identified by cardiotocography, 20 by ultrasound assessment of fetal growth, liquor volume and umbilical artery Doppler, and a further 24 by low hPL in the absence of other abnormality. hPL values less than 1 standard deviation below the mean value for any given gestation was associated with a odds ratio for poor pregnancy outcome of 4.91 (95% CI, 2.8–8.67).

## Discussion

These data confirm the link between RFM and increased incidence of pregnancy complications including SGA, FGR and fetal hypoxia as shown in earlier studies [6], [7], [20]. We have shown that poor pregnancy outcome after RFM is independently related to diastolic blood pressure, estimated fetal weight centile determined by ultrasound biometry and maternal serum hPL levels. These data don't provide evidence in favour of a link between poor perinatal outcome and past obstetric history or recurrent episodes of RFM as seen previously [7]; although with the small number of events there is limited power to detect these particular effects in this study.

It is hypothesised that RFM is linked to FGR and stillbirth as a clinical manifestation of the fetus reacting to nutrient and oxygen deprivation secondary to placental insufficiency [21]. Pregnancies with RFM have altered placental structure and function, including: increased infarction, increased density of syncytial knots, reduced vascularity, a reduction in the syncytiotrophoblast area and reduced neutral amino acid transport [22]. Therefore, the relationship between diastolic blood pressure, estimated fetal weight centile, log [hPL] and poor perinatal outcome after RFM is likely to relate to their association with placental dysfunction. Epidemiological links between elevated blood pressure with placental dysfunction are well established [23], while estimated fetal weight provides the most accurate means to detect the downstream effects of placental function on fetal size. Furthermore, hPL, a placentally-derived hormone, is reduced in maternal serum in pregnancies complicated by FGR [19]. Importantly, other factors manufactured by the syncytiotrophoblast, the cell layer of the placenta at the fetal-maternal interface, such as hCG and progesterone, were also reduced in pregnancies ending in poor perinatal outcome and these differences are related to functional placental mass rather than alterations in gene transcription. Further evidence of placentally-derived factors relating to pregnancy outcome such as progesterone metabolites was seen in a metabolomic analysis of 40 cases and 40 controls derived from this cohort [24].

The association between hPL and poor pregnancy outcome does not necessarily indicate that this marker will have clinical value, but it does suggest that novel placental function tests merit further investigation in the clinical management of RFM. Recently, a similar strategy using placental growth factor (PlGF) has been successfully employed to differentiate babies that have pathological FGR from those who are constitutionally small in the third trimester of pregnancy [25]. Further studies are ongoing to determine whether placentally derived factors such as hPL or PlGF may have clinical utility in determining outcome after presentation with RFM.

This prospective study is limited by the absence of stillbirths from the observed perinatal outcomes. Importantly, four infants were delivered by emergency Caesarean section for fetal compromise. If this intervention had not been employed these infants would likely have died *in utero*. Due to its relative infrequency it is difficult to undertake a study such as this which is powered to detect stillbirth, as over 1,000 women would be required to recruit 10 with stillbirth (Background risk 1∶200, Odds ratio of stillbirth after RFM ∼2). Therefore, prior to commencing the study a composite poor perinatal outcome was determined to include infants that were SGA, born before 37 weeks or admitted to the NICU with perinatal asphyxia. These outcomes were chosen as previous studies found that infants stillborn after RFM were all SGA [7], and infants subject to severe intra-uterine compromise might not die but instead be delivered prematurely or neonatal intensive care [12].

This study did not employ a standard definition of RFM because there is no evidence-based definition of RFM that performs better than maternal perception of RFM alone [11]. In addition, we aimed to undertake a pragmatic study that may be generalised to clinical practice, in which 6–15% of women present with RFM in the third trimester of pregnancy even in the absence of specified alarm limits for fetal activity [10], [12]. The ability of this study to be generalised to a wider population is strengthened by the inclusion of a multi-ethnic population which is representative of the wider population in the North-West of England. Furthermore, this study was prospective with very few losses to follow-up, meaning that the possibility of selection bias was minimised.

The finding that estimated fetal weight centile has the strongest association with poor perinatal outcome following RFM supports the findings of a care-improvement project in Norway in which ultrasound assessment of fetal size and liquor volume in women with RFM was associated with a reduction in stillbirth rate [26], [27]. This reduction in stillbirth was most likely due to the increased detection of infants most at risk of stillbirth i.e. those who were SGA or who had oligohydramnios. The relationship between abnormal fetal heart rate trace and poor pregnancy outcome emphasises the importance of cardiotocography to identify fetuses in immediate danger of stillbirth after presentation with RFM, although these are a small proportion (1.3% in this study) of the total population. This study also agrees with a retrospective cohort study of 524 infants who found that an abnormal, but not pathological CTG are also at increased risk of poor pregnancy outcomes [28]. Therefore, we propose that all women who present with RFM after 28 weeks gestation should have CTG and have an ultrasound assessment of fetal weight and liquor volume.

Nevertheless, a more efficient strategy is needed to predict which pregnancies are most at risk of stillbirth after maternal perception of RFM as a single ultrasound scan has a sensitivity of 33.3% to detect SGA and CTG can give false reassurance which may increase perinatal mortality in low-risk populations [16], [29]. Further studies are needed to test whether incorporation of novel measurements of placental structure and/or function such as hPL or PlGF can better predict poor perinatal outcomes after RFM. Ultimately, these need to be tested in a prospective manner to determine whether intervention for women directed by the testing strategy reduces stillbirth [30]. A pilot study to determine the feasibility and acceptability of a trial of ultrasound fetal assessment in combination with evaluation of placental function to direct the management of RFM is underway (ISCRTN07944306).

## Supporting Information

Table S1 **Data fields collected for prospective cohort study of women presenting with reduced fetal movements.**
(DOC)
