# Supplementary material for: Predictors of Poor Perinatal Outcome following Maternal Perception of Reduced Fetal Movements – A Prospective Cohort Study
Source: PLoS One. 2012 Jul 11;7(7):e39784. doi: 10.1371/journal.pone.0039784 (PMC3394759; doi:10.1371/journal.pone.0039784)
Supplement: Table S1 — Data fields collected for prospective cohort study of women presenting with reduced fetal movements. (DOC) [file pone.0039784.s001.doc]

Table S1 - Data fields collected for prospective cohort study of women presenting with reduced fetal movements

| *Maternal Characteristics* |
| --- |
| Age |
| Height (cm) |
| Weight (kg) |
| Gravidity |
| Parity |
| Ethnicity |
| Smoking (cigarettes/day) |
| Alcohol (units/week) |
| Substance misuse |
| Prescribed Drugs |
| Family History |
| Past Medical History |
| Past Obstetric History |
|  |
| *Presentation with Reduced Fetal Movements* |
| Gestation at presentation |
| Duration of reduced fetal movements |
| Absence of fetal movements |
| Current Obstetric Problems (e.g. preeclampsia / gestational diabetes / fetal growth restriction) |
| Symphysiofundal height (cm) |
| Systolic BP (mmHg) |
| Diastolic BP (mmHg) |
| Urinalysis |
| Further episodes of reduced fetal movements |
| Obstetric Problems before the end of pregnancy |
|  |
| *Cardiotocograph assessment including:* |
| Uterine Activity (contractions/10 minutes) |
| Baseline (beats per minute) |
| Variability (beats per minute) |
| Accelerations |
| Decelerations (Early/Late/Variable) |
| Number of fetal movements felt while on cardiotocography recording |
| Duration of cardiotocography recording |
|  |
| *Ultrasound assessment* |
| Abdominal circumference |
| Head circumference |
| Femur length |
| Estimated fetal weight |
| Estimated fetal weight centile |
| Amniotic fluid index |
| Maximum pool depth |
| Umbilical Artery Doppler Pulsatility Index |
|  |
| *Pregnancy Outcome* |
| Live or Stillborn |
| Gestation at delivery |
| Gender |
| Birthweight |
| Customised birthweight centile |
| Placental Weight |
| Mode of Delivery |
| Was labour induced |
| Was labour augmented |
| Evidence of meconium liquor |
| Apgar score at 1 minute |
| Apgar score at 5 minutes |
| Arterial pH |
| Arterial Base Excess |
| Venous pH |
| Venous Base Excess |
| Admission to NICU |
